# Aberrant brain functional connectivity in patients with dysthyroid optic neuropathy: a resting-state fMRI study

**DOI:** 10.3389/fnins.2026.1935362

**Published:** 2026-09-21

**Authors:** Jin-Ling Lu, Yu-Hui Zhou, Huan-Huan Chen, Ying Wan, Jiang Zhou, Xiao-Quan Xu, Hao Hu, Fei-Yun Wu

**Affiliations:** 1 Department of Radiology, The First Affiliated Hospital With Nanjing Medical University, Nanjing, China; 2 Department of Endocrinology, The First Affiliated Hospital With Nanjing Medical University, Nanjing, China

**Keywords:** degree centrality, dynamic analysis, dysthyroid optic neuropathy, resting-state fMRI, voxel-mirrored homotopic connectivity

## Abstract

**Objectives:**

We aimed to investigate the alterations of static and dynamic brain functional connectivity in dysthyroid optic neuropathy (DON) using resting-state functional MRI (rs-fMRI) with the voxel-mirrored homotopic connectivity (VMHC) and degree centrality (DC) methods.

**Materials and methods:**

Seventy-five thyroid-associated ophthalmopathy (TAO) patients (29 DON and 46 non-DON) and 32 healthy controls (HCs) were prospectively recruited. Static and dynamic VMHC (sVMHC and dVMHC) and DC (sDC and dDC) values were measured and compared among groups. Support-vector machine (SVM) classification method was employed to examine the diagnostic performance of identified models in distinguishing DON patients from non-DON patients.

**Results:**

Compared to non-DON patients, DON patients showed decreased sVMHC in cerebellar lobule VI, inferior occipital gyrus (IOG) and superior occipital gyrus (SOG), alongside decreased dVMHC in IOG. DON patients also exhibited decreased sDC in bilateral middle occipital gyrus (MOG) in comparison to non-DON patients. Meanwhile, DON patients had lower sVMHC in lingual gyrus and MOG, together with decreased sDC in left MOG compared to HCs. Additionally, dVMHC in IOG of DON patients was positively correlated with the quality-of-life scores for appearance (*r* = 0.419, *p* = 0.033). When detecting DON, combined model (sVMHC+dVMHC+sDC) showed best diagnostic performance (AUC = 0.9528), followed by the dVMHC model (AUC = 0.9378), sDC model (AUC = 0.8598), and sVMHC model (AUC = 0.8193).

**Conclusion:**

Brain functional connectivity differed between TAO patients with and without DON, which may reflect the underlying neural mechanism of disease. The dVMHC index showed promising discriminative potential for identifying DON. Combining static and dynamic metrics could further optimize diagnostic efficiency.

## 1 Introduction

Dysthyroid optic neuropathy (DON) is the most severe complication of thyroid-associated ophthalmopathy (TAO). It can cause permanent vision loss and substantially diminish patients’ quality of life. Although its full pathogenesis remains unestablished, the widely accepted mechanism is orbital apex crowding syndrome, which leads to optic nerve compression and mechanical stretching. The typical clinical manifestations of DON are impaired visual functions, such as decreased visual acuity, color vision dysfunction, visual field defects, and optic disc edema. However, these symptoms may also manifest in non-DON patients, which may preclude accurate diagnosis of DON in the early stage and subsequently result in unsatisfactory improvement of visual functions after therapy. Therefore, a better understanding of the neurobiological mechanisms of DON and identification of early detection means are clinically crucial to prevent irreversible vision loss.

Several studies have reported structural and functional changes in the brain of DON patients. Wu et al. (2023) found that DON patients exhibited significantly decreased gray matter volume in vision-related brain regions. A diffusion-tensor imaging study detected microstructural impairment in the entire visual pathway in DON (Liu et al., 2023a). Resting-state functional MRI (rs-fMRI) is a non-invasive neuroimaging technique to investigate spontaneous brain activity that has recently been utilized in the study of DON. Liu et al. (2023b) discovered that the decreased regional homogeneity value in the superior temporal gyrus in DON patients was associated with visual dysfunction. Another rs-fMRI study using the amplitude of the low-frequency fluctuations method concluded that the observed anomalies in the visual cortex in DON patients were closely correlated with the presence of optic nerve compression (Jiang et al., 2021). In the research of DON, a gap exists because previous studies have mainly concentrated on the alterations of localized functional activity. Although intrinsic local metrics offer insights into regional neural activity, they have limitations in capturing inter-regional communication dynamics (Rezaei et al., 2022). Functional connectivity analyses, which focus on the interactions between different brain regions and assess the brain as an integrated network, can provide essential information about how information processing is globally disrupted (Lv et al., 2018). To our knowledge, there is a lack of research on brain functional connectivity in DON patients.

The voxel-mirrored homotopic connectivity (VMHC) method quantifies the homotopic connectivity between each voxel in one hemisphere and its mirror voxel counterpart in the opposite hemisphere (Zuo et al., 2010). VMHC has been proven to be useful in detecting the functional connectivity between interhemispheric regions in various ocular and neural diseases, such as amblyopia, blindness, and Parkinson’s disease (Peng et al., 2021; Shao et al., 2018; Zhang et al., 2022). Degree centrality (DC) is a graph theory-based local metric. It measures the number of connections of a target voxel, indicating the significance of that node in the brain network. DC has been applied to investigate altered brain activity in patients with diabetic optic neuropathy, neuromyelitis optica spectrum disorder, and glaucoma (Chen et al., 2022; Wei et al., 2022; Xu et al., 2020). Wu et al. (2023) demonstrated that DON patients had significantly decreased DC values in Brodmann areas 17 and 18, which may provide insights into the underlying mechanism of vision loss. In summary, more studies on functional connectivity in DON are required.

Moreover, as spontaneous neural activity is dynamic in the time dimension (Preti et al., 2017), the traditional static indices may ignore the dynamic features of brain connectivity. Dynamic properties have been proven to exhibit good reliability and reproducibility (Guo et al., 2019). Past research based on dynamic analysis has shown altered brain activity and connectivity in TAO and suggested areas of further investigation regarding its potential neural mechanisms (Jiang et al., 2023). A recent study suggested that the dynamic amplitude of low-frequency fluctuation may serve as a potential biomarker for diagnosing DON (Lu et al., 2024). Quantifying the time-varying functional connectivity may provide a better strategy for exploring the fundamental properties of brain activity in DON.

To address some of the existing research gaps, this study examined both the static and dynamic alterations of functional connectivity in DON using the rs-fMRI–based VMHC and DC methods. The support vector machine (SVM) method was applied to classify DON and non-DON cohorts based on the VMHC and DC values extracted from the abnormal brain areas.

## 2 Materials and methods

### 2.1 Subjects

This study received approval from our institutional review board. All subjects volunteered to participate in the study and signed the informed consent form. A total of 29 DON patients and 46 non-DON patients were prospectively recruited from our hospital. During the same period, 32 healthy controls (HCs) were recruited from the local community. TAO was diagnosed using the Bartley criteria. DON was defined by a history of TAO and at least any two of the following clinical manifestations: (1) best-corrected visual acuity (BCVA) < 0.8; (2) visual field defects; (3) relative afferent pupillary defects; (4) optic disc edema; and (5) impairment of color vision. Exclusion criteria were as follows: (1) any evidence of other ophthalmologic diseases; (2) history of eye surgery; (3) history of neurological or psychiatric disorders; (4) contraindications for MRI examination; or (5) history of radiotherapy or systemic glucocorticoid therapy in the past 3 months.

### 2.2 Clinical assessment

Demographic and clinical variables were acquired from the participants. Disease activity was assessed using the modified 7-point clinical activity score (CAS; Bartalena et al., 2021). The duration of TAO was determined from the onset of relevant ophthalmic symptoms to the date of the MRI scan. The proptosis test, BCVA test and visual field test were conducted. Serum levels of free triiodothyronine (FT3), free thyroxine (FT4), and thyrotropin (TSH) were collected. Montreal Cognitive Assessment (MoCA), Hamilton Anxiety Rating Scale (HARS), and Hamilton Depression Rating Scale (HDRS) scores were collected. In addition, a quality of life (QoL) questionnaire, focusing on visual function and appearance, was completed by each patient (Lin et al., 2015).

### 2.3 MRI data acquisition

MRI examinations were conducted using a 3.0-T MRI system (Magnetom Skyra; Siemens Healthcare) with a 20-channel head and neck coil. High-resolution sagittal structural T1-weighted images (T1WIs) were obtained using an MPRAGE sequence with the following parameters: field of view (FOV) = 256 mm × 256 mm, repetition time (TR) = 1900 ms, echo time (TE) = 2.45 ms, flip angle = 9°, number of slices = 176, thickness = 1.0 mm, matrix = 256 × 256, and voxel size = 1 mm × 1 mm × 1 mm. Then, functional images were acquired axially by applying an echo planar imaging sequence with the following scanning parameters: FOV = 240 mm × 240 mm, TR = 2000 ms, TE = 30 ms, flip angle = 90°, number of slices = 35, thickness = 4.0 mm, matrix = 64 × 64, and voxel size = 3.75 mm × 3.75 mm × 4 mm. The total scanning time was 12 min 26 s.

### 2.4 Data preprocessing

All rs-fMRI data were preprocessed by applying the DPABI toolbox^1^ (Yan et al., 2016) based on SPM 12.^2^ The first 10 images of each dataset were removed to ensure magnetization stability. Slice timing correction and realignment for head motion correction were performed on the remaining 230 consecutive images. Participants with head motion of >2.5 mm of translation or 2.5° of rotation were excluded. Besides, we calculated the mean frame-wise displacement (FD; Power et al., 2012) and included it as a covariate in the group-level analysis to minimize the impact of head motion. The images were then spatially normalized to the Montreal Neurological Institute (MNI) template (resampling voxel size = 3 mm × 3 mm × 3mm) using T1 image unified segmentation and smoothed with a 6 mm full-width at half-maximum (FWHM) Gaussian kernel. Nuisance covariates, including the Friston-24 parameters of head motion, white matter signal, cerebrospinal fluid signal, and the brain’s global signal, were removed by linear regression. Finally, linear detrending and temporal bandpass filtering (frequency range of 0.01–0.08 Hz) were performed on the data.

### 2.5 Static VMHC and static DC analyses

Static VMHC (sVMHC) and static DC (sDC) analyses were performed using the DPABI software. For sVMHC calculation, first, a mean image was created by averaging all subjects’ spatially normalized T1WIs. Second, a group-specific symmetric template was generated by averaging this image with its flipped version. The normalized T1WIs were then registered to the symmetric template and applied to the non-linear transformation to the normalized functional images. Finally, for each subject, the homotopic connectivity was calculated as Pearson’s correlation between each pair of mirrored voxel time series. To increase the normality of the distribution, Fisher r-to-z transformation was performed for the correlation coefficients, and the sVMHC z-maps were used for the subsequent analyses.

To avoid potential inflation of DC values caused by spatial smoothing, preprocessed data without smoothing were used for the DC analysis. We restricted DC calculation to positive correlations above the threshold of *r* = 0.25 to eliminate weak correlations. The individual level DC was then normalized to a z-score map for standardization purpose, followed by smoothing with a 6 mm FWHM Gaussian kernel.

### 2.6 Dynamic VMHC and dynamic DC analyses

The analyses of dynamic VMHC (dVMHC) and dynamic DC (dDC) were carried out with the Temporal Dynamic Analysis (TDA) toolkits of DPABI, which are based on a sliding-window approach. In this study, a sliding-window length of 50 TR (100 s) and a shifting step size of 1 TR (2 s) were used to optimize the balance between the ability to resolve dynamics and the quality of connectivity estimation. The remaining 230 time points after removing the first 10 time points were segmented into 181 windows in total. In each sliding window, VMHC and DC were calculated by the same method used in the computation of sVMHC and sDC. The standard deviation of the *z*-values at each voxel across all windows was calculated to depict the dVMHC and dDC. Finally, the dDC map was smoothed with a 6 mm FWHM Gaussian kernel.

### 2.7 Statistical analyses

Demographic and clinical data were analyzed using SPSS (version 26, IBM SPSS Inc.). To compare the three groups, analysis of covariance (ANCOVA) and Kruskal–Wallis test were used for the continuous variables with normal distribution and the variables with non-normal distribution, respectively. To compare the two groups, the independent-sample *t*-test was used for the normally distributed continuous variables, and the Mann–Whitney U-test was used for the non-normally distributed variables. The χ^2^ test was applied for the categorical variables. The threshold for statistical significance was set at *p* < 0.05 (two-tailed).

For the imaging data, namely, the sVMHC, sDC, dVMHC, and dDC values, ANCOVAs were performed to determine the differences among the three groups within a gray-matter mask with age, gender, education, and mean FD as the covariates. Subsequently, these regions were extracted as masks, and the two-sample post-hoc *t*-test was performed between each pair of groups. Gaussian Random Field (GRF) correction was utilized for multiple comparison correction (voxel-level *p* < 0.001, cluster-level *p* < 0.05, two-tailed). Partial correlation analyses, with sex, age, and education level as covariates, were used to assess the relationships between the rs-fMRI metrics, which indicated significant alterations in the DON group and the clinical variables.

### 2.8 SVM analyses

Support-vector machine was used to determine the ability of the rs-fMRI values extracted from the abnormal brain regions to differentiate between the DON and non-DON groups. It was performed by utilizing the LIBSVM software in MATLAB. Four rs-fMRI models were built: (1) sVMHC; (2) dVMHC; (3) sDC; and (4) combined (sVMHC + dVMHC + sDC) models. Leave-one-out cross-validation was performed. Receiver operating characteristic (ROC) curves were created for each model. DeLong’s test was used to compare the area under the curves (AUCs) of the machine learning models. Statistical significance of the classification accuracy was evaluated using the non-parametric permutation tests (5000 times).

### 2.9 Validation analyses

To confirm our main findings of the dVMHC alterations, two different sliding-window lengths [30 TR (60 s) and 70 TR (140 s)] were used to perform further validation analyses.

## 3 Results

### 3.1 Demographic and clinical characteristics

The participants’ demographic and clinical data are listed in Table 1. There were no significant differences in age, gender, years of education, or smoking history among the three groups. Both the DON and non-DON groups showed significantly lower MoCA scores (both *p* ≤ 0.001) compared with the HCs, along with higher HDRS and HARS scores (all *p* < 0.001). Compared with the non-DON patients and HCs, the DON patients showed significantly lower BCVA (both *p* < 0.001) in both eyes. Between-group comparisons found that the DON patients had higher HDRS and HARS scores, proptosis, and CAS, and lower QoL scores for visual functioning and appearance as well as lower MoCA, scores than the non-DON group (all *p* < 0.05). There were no significant differences in TAO duration or serum levels of FT3, FT4, and TSH between the DON and non-DON groups.

**TABLE 1:** Demographic and clinical characteristics.

Items	DON (*n* = 29)	Non-DON (*n* = 46)	HC (*n* = 32)	*P*
Age (years)	56.79 ± 11.45	54.15 ± 8.08	52.44 ± 9.36	0.201
Gender (male/female)	12/17	17/29	13/19	0.913
Education level (years)	9.59 ± 3.51	10.37 ± 3.54	10.34 ± 3.07	0.580
Handedness (right/left)	29/0	46/0	32/0	>0.999
TAO duration (months)	11.10 ± 8.98	12.11 ± 12.46		0.789
Smoking history (yes/no)	11/18	22/24	15/17	0.677
CAS (OD)	4.41 ± 1.21	2.26 ± 1.04		<0.001
CAS (OS)	4.34 ± 1.23	2.24 ± 1.04		<0.001
FT3 (pmol/L)	5.31 ± 1.56	6.32 ± 5.64		0.777
FT4 (pmol/L)	16.57 ± 5.38	18.45 ± 13.14		0.728
TSH (mlU/L)	2.42 ± 6.70	1.94 ± 2.98		0.143
BCVA (OD)	0.60 ± 0.29	0.96 ± 0.12	1.00 ± 0.12	<0.001
BCVA (OS)	0.58 ± 0.28	0.95 ± 0.15	1.00 ± 0.11	<0.001
VFI (OD)	0.72 ± 0.31			
VFI (OS)	0.78 ± 0.29			
Proptosis (OD) (mm)	20.83 ± 3.19	18.63 ± 2.97		0.006
Proptosis (OS) (mm)	21.41 ± 3.35	18.58 ± 3.03		0.001
Total score of QoL				
Visual functioning	42.86 ± 19.65	60.58 ± 29.89		0.003
Appearance	61.42 ± 20.05	66.58 ± 19.42		0.368
Total score of HDRS	13.24 ± 6.41	9.26 ± 4.54	2.78 ± 1.93	<0.001
Total score of HARS	13.97 ± 6.34	9.91 ± 6.26	3.16 ± 1.59	<0.001
Total score of MoCA	23.93 ± 3.52	26.02 ± 3.83	29.03 ± 0.97	<0.001

Data are presented as mean ± standard deviation. BCVA, best-corrected visual acuity; CAS, clinical activity score; DON, dysthyroid optic neuropathy; FT3, free triiodothyronine; FT4, free thyroxine; HARS, Hamilton Anxiety Rating Scale; HC, healthy control; HDRS, Hamilton Depression Rating Scale; MoCA, Montreal Cognitive Assessment; OD, oculus dexter; OS, oculus sinister; QoL, quality of life; TAO, thyroid-associated ophthalmopathy; TSH, thyrotropin; VFI, visual field index.

### 3.2 Static and dynamic VMHC values among groups

The ANCOVA results showed significant differences in sVMHC among the three groups, primarily in cerebellar lobule VI, the inferior occipital gyrus (IOG), and the superior occipital gyrus (SOG) (Figure 1 and Supplementary Table 1). In the pairwise comparisons, the DON group exhibited decreased sVMHC in cerebellar lobule VI, IOG, and SOG compared with the non-DON group. Compared with the HCs, the DON group exhibited decreased sVMHC in the lingual gyrus (LING) and middle occipital gyrus (MOG) (Figure 1 and Table 2).

**FIGURE 1:**
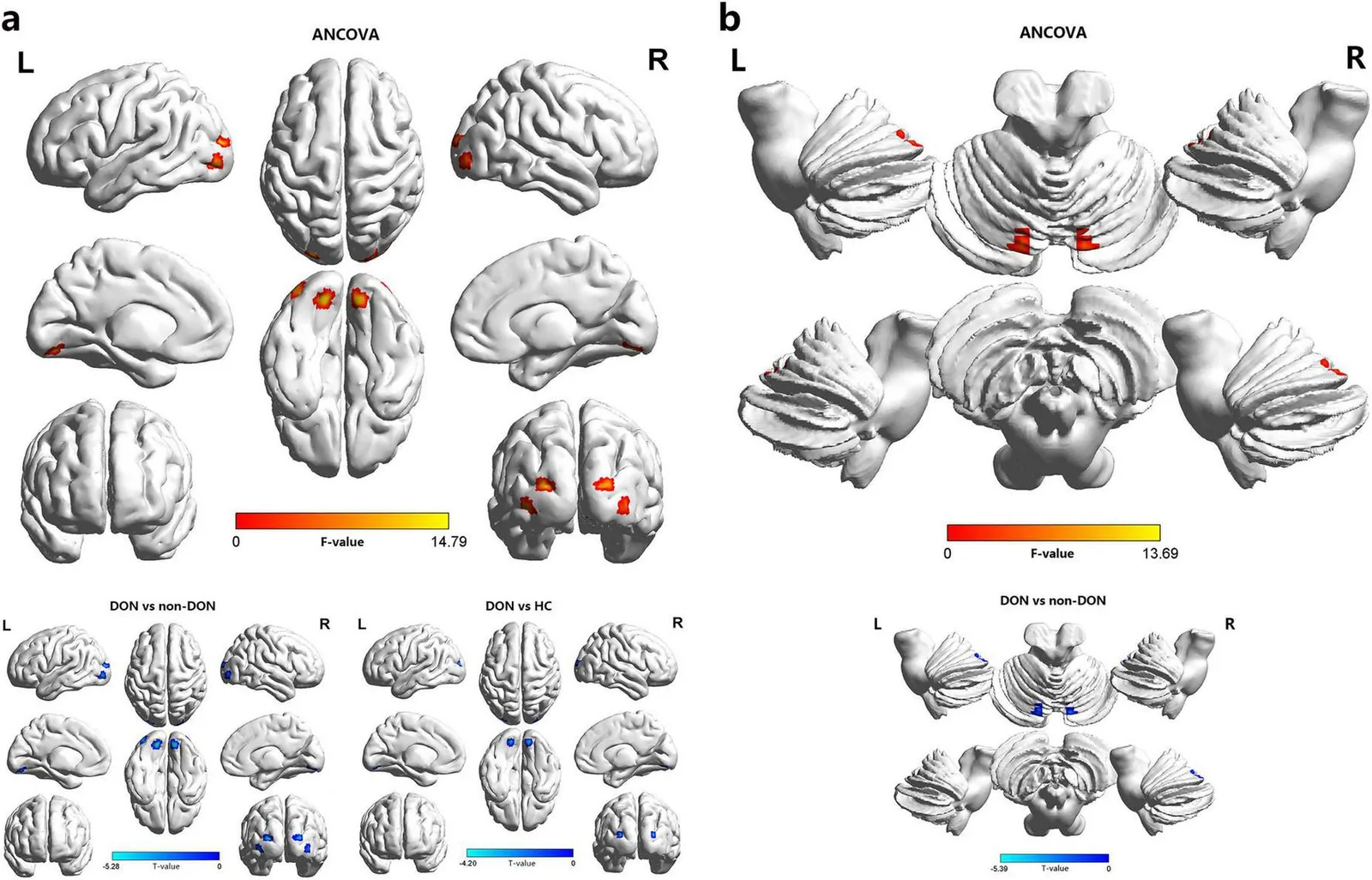
Brain regions with significant sVMHC differences among DON, non-DON, and HC groups in the cerebrum **(a)** and cerebellum **(b)** (voxel level: *p* < 0.001, cluster level: *p* < 0.05, GRF corrected). In the ANCOVA results, red denotes regions that differed among the three groups. In the post-hoc pairwise comparison results, cold colors indicate decreased sVMHC values. ANCOVA, analysis of covariance; DON, dysthyroid optic neuropathy; HC, healthy controls; L, left; R, right; sVMHC, static voxel-mirrored homotopic connectivity.

**TABLE 2:** Brain areas showing significant differences in sVMHC and dVMHC values across three groups.

Conditions	Brain region	BA	Cluster	MNI coordinates			*T*-values
			size	X	Y	Z	
sVMHC							
DON vs. non-DON	R/L Cerebellar lobule VI	/	27	±12	−81	−15	−5.3931
	R/L IOG	19	34	±33	−84	−3	−5.2814
	R/L SOG	18	27	±21	−93	9	−5.0816
DON vs. HCs	R/L LING	18	11	±15	−78	−12	−4.1998
	R/L MOG	18	9	±24	−93	15	−3.6596
dVMHC							
DON vs. non-DON	R/L IOG	19	10	±51	−72	−9	−7.6527
Non-DON vs. HCs	R/L IOG	19	6	±51	−75	−9	5.1244

GRF correction, voxel level: *p* < 0.001, cluster level: *p* < 0.05. BA, Brodmann’s areas; DON, dysthyroid optic neuropathy; dVMHC, dynamic voxel-mirrored homotopic connectivity; HC, healthy controls; IOG, inferior occipital gyrus; L, left; LING, lingual gyrus; MNI, Montreal Neurological Institute; MOG, middle occipital gyrus; R, right; SOG, superior occipital gyrus; sVMHC, static voxel-mirrored homotopic connectivity.

In terms of the dVMHC analysis, the ANCOVA results showed significant dVMHC differences in the IOG among the three groups (Figure 2 and Supplementary Table 1). In the pairwise comparisons, the DON group exhibited decreased dVMHC in the IOG compared with the non-DON group. Moreover, the non-DON group showed higher dVMHC in the IOG compared with the HCs (Figure 2 and Table 2).

**FIGURE 2:**
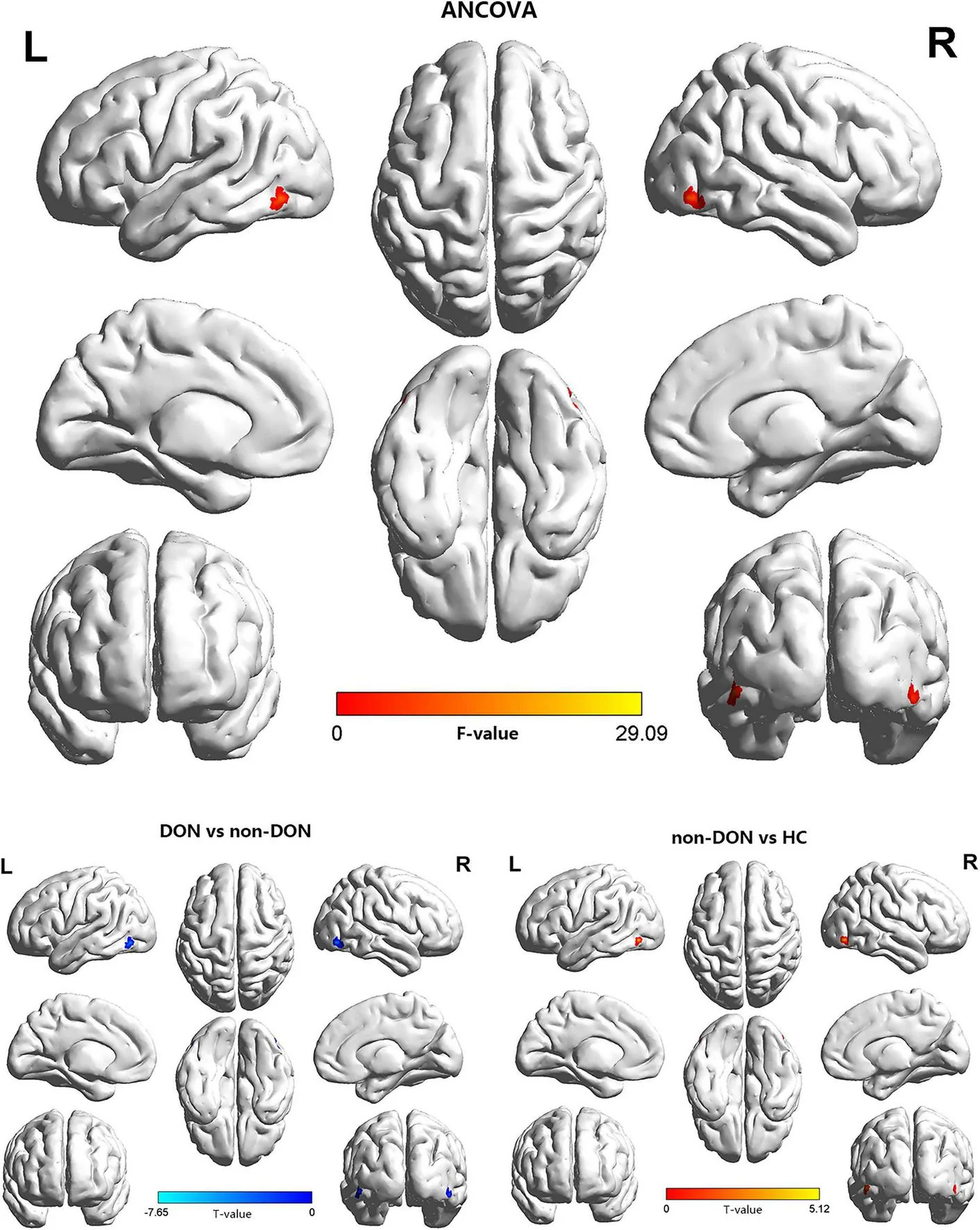
Brain regions with significant dVMHC differences among DON, non-DON, and HC groups (voxel level: *p* < 0.001, cluster level: *p* < 0.05, GRF corrected). In the ANCOVA results, red denotes regions that differed among the three groups. In the post-hoc pairwise comparison results, warm and cold colors indicate increased and decreased dVMHC values, respectively. ANCOVA, analysis of covariance; DON, dysthyroid optic neuropathy; dVMHC, dynamic voxel-mirrored homotopic connectivity; HC, healthy controls; L, left; R, right.

### 3.3 Static and dynamic DC values among the groups

The ANCOVA results showed significant sDC differences among the three groups, primarily in the bilateral MOG (Figure 3 and Supplementary Table 1). In the pairwise comparisons, compared with the non-DON group, the DON group exhibited decreased sDC in the bilateral MOG. Compared with the HCs, the DON group showed decreased sDC in the left MOG (Figure 3 and Table 3).

**FIGURE 3:**
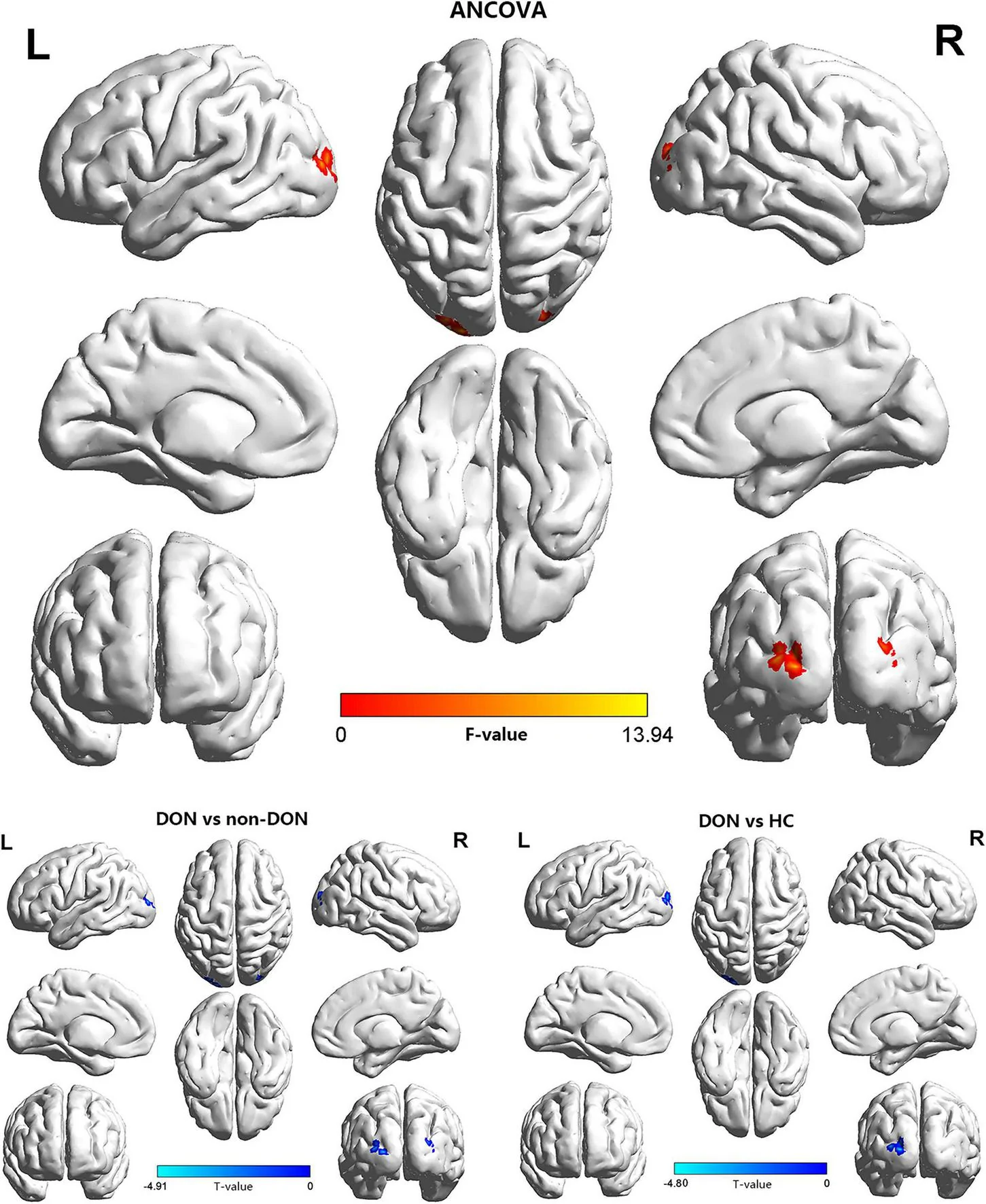
Brain regions with significant sDC differences among DON, non-DON, and HC groups (voxel level: *p* < 0.001, cluster level: *p* < 0.05, GRF corrected). In the ANCOVA results, red denotes regions that differed among the three groups. In the post-hoc pairwise comparison results, cold colors indicate decreased sDC values. ANCOVA, analysis of covariance; DON, dysthyroid optic neuropathy; HC, healthy controls; L, left; R, right; sDC, static degree centrality.

**TABLE 3:** Brain areas showing significant differences in sDC values across three groups.

Conditions	Brain region	BA	Cluster	MNI coordinates			*T*-values
			size	X	Y	Z	
sDC							
DON vs. non-DON	L MOG	18	14	−27	−93	12	−4.6107
	R MOG	18	10	30	−90	15	−4.9142
DON vs. HCs	L MOG	18	14	−27	−93	6	−4.7952

GRF correction, voxel level: *p* < 0.001, cluster level: *p* < 0.05. BA, Brodmann’s areas; DON, dysthyroid optic neuropathy; HC, healthy controls; L, left; MNI, Montreal Neurological Institute; MOG, middle occipital gyrus; R, right; sDC, static degree centrality.

In the dDC analysis, the DON group showed no significant differences compared with the non-DON group and HCs.

### 3.4 Correlation analysis

In the DON group, the dVMHC in the IOG was positively correlated with the QoL scores for appearance (*r* = 0.419, *p* = 0.033) (Figure 4). No significant correlations were found between the rs-fMRI metrics and other clinical measures.

**FIGURE 4:**
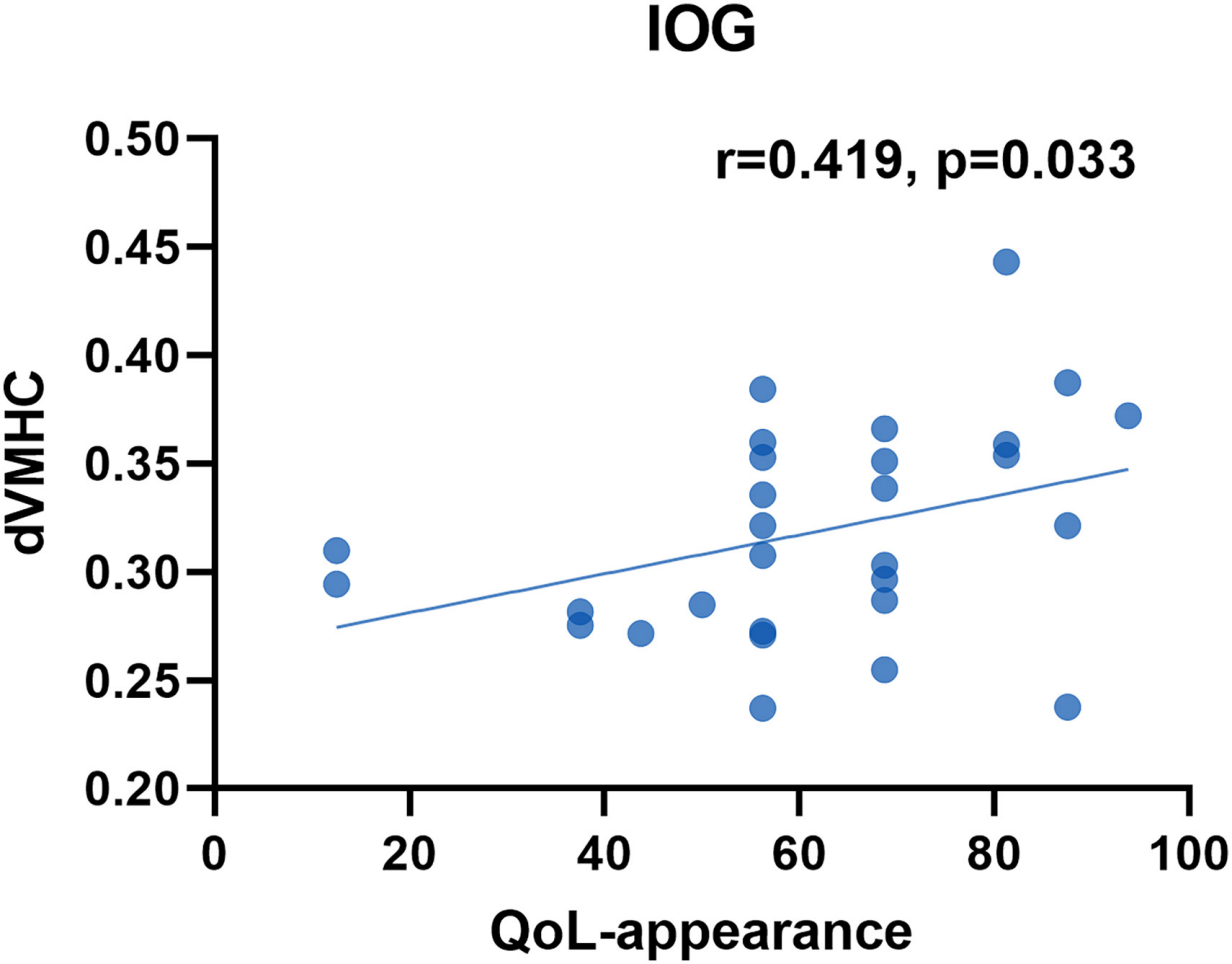
Scatter diagrams showing significant correlations between clinical characteristics and the dVMHC values in DON patients. dVMHC in IOG was positively correlated with the QoL scores for appearance (*r* = 0.419, *p* = 0.033). dVMHC, dynamic voxel-mirrored homotopic connectivity; IOG, inferior occipital gyrus.

### 3.5 SVM classification results

The combined model (sVMHC + dVMHC + sDC) exhibited the best diagnostic performance (AUC 0.9528, accuracy 92%, sensitivity 86.21%, and specificity 97.83%), followed by the dVMHC model (AUC 0.9378, accuracy 86.67%, sensitivity 82.76%, and specificity 97.83%), the sDC model (AUC 0.8598, accuracy 77.33%, sensitivity 82.76%, and specificity 78.26%), and the sVMHC model (AUC 0.8193, accuracy 77.33%, sensitivity 79.31%, and specificity 84.78%). The ROC curves of the different rs-fMRI models are shown in Figure 5.

**FIGURE 5:**
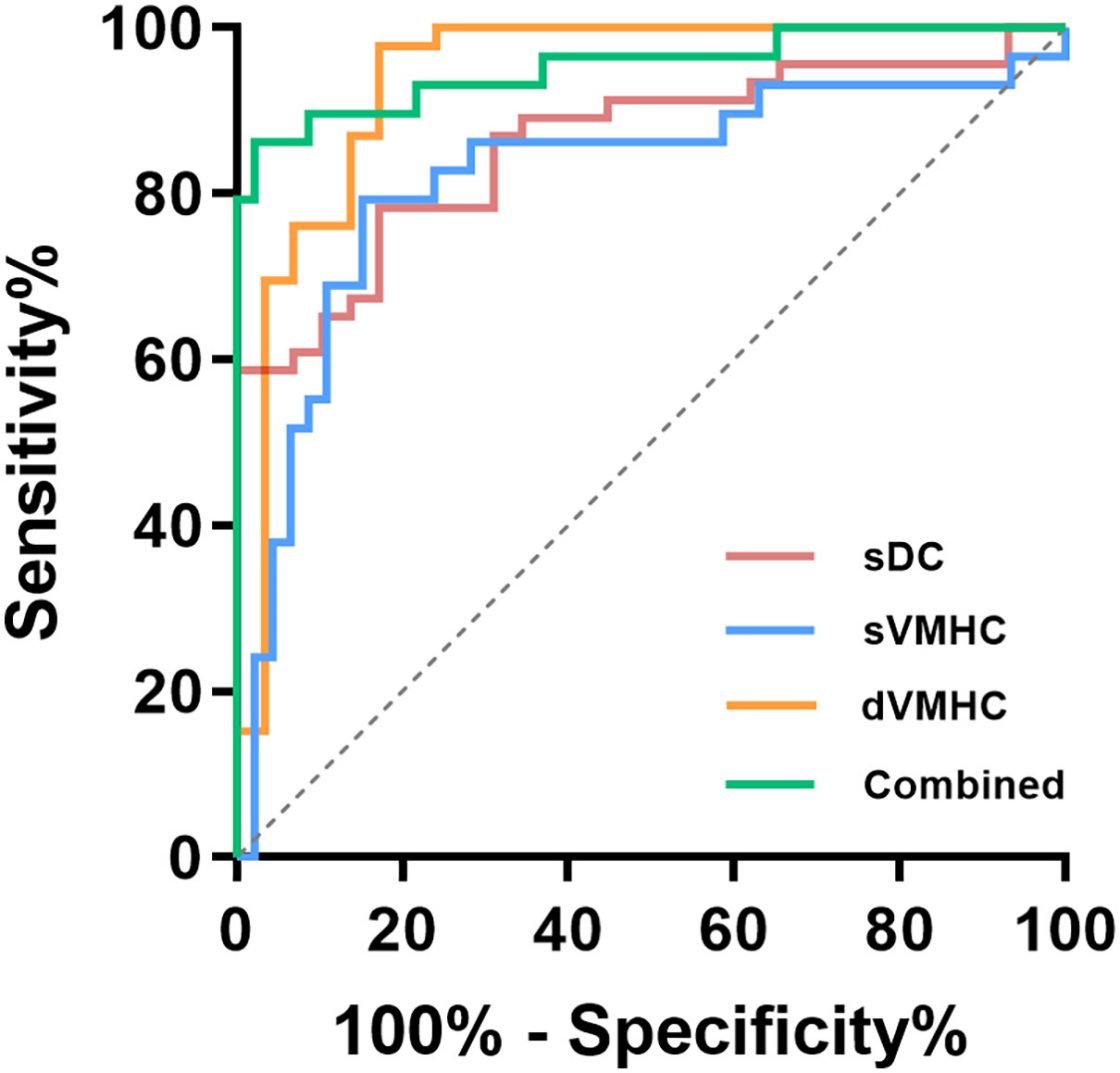
Receiver operating characteristic curves of the SVM-based classifiers. Combined, sDC + sVMHC + dVMHC; dVMHC, dynamic voxel-mirrored homotopic connectivity; sDC, static degree centrality; sVMHC, static voxel-mirrored homotopic connectivity; SVM, support vector machine.

### 3.6 Validation results

Analyses using different sliding-window lengths (30 TRs and 70 TRs) supported our main findings of the dVMHC alterations (Supplementary Figure 1 and Supplementary Table 2).

## 4 Discussion

Our study investigated the static and dynamic brain functional connectivity alterations in DON using VMHC and DC analyses, leading to two main findings: (1) The DON group exhibited significantly decreased sVMHC in cerebellar lobule VI, along with decreased sVMHC, dVMHC, and sDC in the visual cortex compared with the non-DON group. (2) Machine learning models constructed using the identified rs-fMRI metrics exhibited strong performance in differentiating DON patients from non-DON patients. Among the four models, the combined model demonstrated the best classification results, underscoring the importance of dynamic analysis. These findings provide novel insights into the neurobiological mechanisms of DON, which may assist in the timely diagnosis and treatment of the disease.

Our data showed decreased sVMHC in the SOG, decreased sVMHC and dVMHC in the IOG, and decreased sDC in the bilateral MOG in the DON patients relative to the non-DON patients. Compared with the HCs, the DON patients had significantly decreased sVMHC values in the LING and MOG. All the above-mentioned brain areas are located in the occipital lobe. It is well established that the occipital area is the core of visual processing and is involved in visual formation and visual perception (Yu et al., 2017). Specifically, the LING is part of the ventral visual stream and functions to process category-related visual information such as color, size, contour, shape, and object recognition (Shao et al., 2019). The MOG is an important component of the dorsal visual stream (DVS; Wandell et al., 2007). The DVS extends to the dorsal prefrontal cortex and processes visuospatial perception (Liu et al., 2017). The IOG, also termed the occipital face area, performs a critical function in the visual processing of faces. Neuroimaging studies have consistently reported heightened activation in this region during facial recognition tasks (Liu et al., 2010; Sergent et al., 1992). Aberrant interhemispheric functional synchronization within these regions, linked to visual dysfunction, has been documented in individuals with comitant exotropia, open globe injury, and amblyopia (Chen et al., 2023; Liang et al., 2017; Ye et al., 2018). In DON, prior research has identified decreased DC values in the primary and secondary visual cortices (Wu et al., 2023). Given the significantly lower bilateral BCVA observed in DON patients, the concomitant alterations in functional connectivity within the occipital regions suggest an association between visual dysfunction and altered cortical functional organization in DON. However, because of the cross-sectional design of the present study, the temporal and causal relationship between these alterations cannot be determined. The observed changes in occipital functional connectivity may reflect consequences or compensatory responses to impaired visual input caused by optic nerve dysfunction, while alternatively, disease-related alterations in cortical functional organization may contribute to impaired visual processing. Longitudinal studies incorporating repeated neuroimaging and detailed visual assessments before and after treatment are warranted to clarify these possibilities.

Our analyses also revealed decreased sVMHC in the cerebellar lobule VI, which is located in the posterior cerebellar lobe (Buckner et al., 2011). The posterior cerebellar lobe is proven to be critical in reflexive and voluntary eye movements (Liu et al., 2019). One previous study indicated that dysfunction of the posterior cerebellar lobe was related to abnormal coordination of the extraocular muscles in patients with concomitant strabismus (Tan et al., 2018). Hence, the observed disrupted interhemispheric functional coordination in the cerebellar lobe in the DON group might also lead to disturbed function of eye movements. Moreover, as a neural hub in the cerebellothalamocortical circuit, lesions in this region may cause cerebellar cognitive affective syndrome, characterized by executive dysfunction, deficits in linguistic skills, and emotional lability (Schmahmann, 2019). In research on hyperthyroidism patients, diminished functional connectivity of the posterior cerebellar lobe was reported to be connected with cognitive impairments and anxiety symptoms (Li et al., 2017). A number of studies have noted that TAO patients not only exhibit ocular symptoms but also may suffer from memory issues, emotional dysregulation, negative behavior, and personality irregularities (Chen W. et al., 2021; Cockerham et al., 2021). Therefore, combined with the lower MoCA scores and higher HDRS and HARS scores in the DON group, we surmised that the detected lower sVMHC values in the cerebellum might also be linked to their emotional and psychiatric symptoms.

Notably, the dVMHC values in the IOG significantly decreased in the DON group compared with the non-DON group. Comparative analyses suggested that the sVMHC may be more sensitive in detecting intergroup neural differences between the DON patients and non-DON patients than the dVMHC, whereas the dVMHC could offer enhanced specificity in identifying the brain areas with significant differences between the DON and non-DON groups compared with the sVMHC. Prior research on subacute stroke patients and TAO patients has also offered the same speculation (Chen W. et al., 2021; Chen W. et al., 2021). Accordingly, the integrated application of static and dynamic analyses may provide enhanced capacity to elucidate the potential neurological mechanism of abnormal functional connectivity in DON. A large proportion of patients with TAO have clinically significant mood disorders. In the present study, correlation analysis indicated that the dVMHC in the IOG of DON patients was positively correlated with the QoL scores for appearance. This result suggests that the ocular symptoms of the condition, such as exophthalmos, periorbital edema, and upper eyelid retraction, may negatively affect facial appearance, thereby contributing to the development of depression and other psychological disturbances. The non-DON patients showed higher dVMHC values in the IOG than those exhibited by the HC group, which may indicate probable functional compensation in the corresponding brain region.

To determine whether the rs-fMRI values of the significant clusters had potential discriminative value for distinguishing DON from non-DON patients, we employed SVM-based machine learning analysis. The results demonstrated superior diagnostic efficacy of the dVMHC union compared with the sVMHC union and sDC union (AUC: 0.9378 vs. 0.8193/0.8598). The model with the combination of sVMHC, dVMHC, and sDC exhibited the optimal diagnostic efficacy (AUC: 0.9528), with higher sensitivity (86.21% vs. 82.76%) than the dVMHC model. According to prior studies, the human brain exhibits highly time-varying properties and maintains a dynamic balance. A growing body of evidence has consistently indicated that dynamic metrics are valuable in distinguishing between patients and HCs (Chen W. et al., 2021; Lu et al., 2024; Tian et al., 2023; Zhang et al., 2023). In the current study, the observed results of the dVMHC reflected the abnormality of dynamic brain functional connectivity in the DON patients, potentially offering novel insights into the neurobiological mechanisms underlying DON. The performance of the dVMHC union underscores the potential of utilizing dynamic analyses in the diagnosis of DON to formulate targeted clinical interventions for patients.

There are some limitations of this study. First, the present study was a cross-sectional study, and the interpretations must be validated through a longitudinal study. Second, the relatively small sample size, particularly the limited number of DON patients, may have increased the risk of overfitting in the SVM analyses and may have resulted in optimistic estimates of classification performance. Therefore, the relatively high AUC values, particularly that of the combined model, should be interpreted cautiously. Larger multicenter cohorts and independent external validation are necessary to determine the reproducibility and generalizability of these findings. Third, future research utilizing multi-modal neuroimaging methods would provide more comprehensive information than a single modality, thereby facilitating a full understanding of the neurological basis of DON.

In conclusion, our study revealed that both static and dynamic brain functional connectivity differed between TAO patients with and without DON, hinting at the underlying neural mechanisms of the disease. The static and dynamic brain functional connectivity metrics, especially the dVMHC, may have potential discriminative value for distinguishing DON from non-DON patients.

## Data Availability

The raw data supporting the conclusions of this article will be made available by the authors, without undue reservation.

## Footnotes

1
https://rfmri.org/DPABI

2
http://www.fil.ion.ucl.ac.uk/spm/
