# Supplementary material for: Aberrant brain functional connectivity in patients with dysthyroid optic neuropathy: a resting-state fMRI study
Source: Front Neurosci. 2026 Sep 21;20:1935362. doi: 10.3389/fnins.2026.1935362 (PMC13635314; doi:10.3389/fnins.2026.1935362)
Supplement: Supplementary file 1 [file Data_Sheet_1.docx]

**Supplementary Materials**

**Table S1.** Brain areas showing significant differences in sVMHC, sDC and dVMHC values across the three groups.

| **Conditions** | **Brain region** | **BA** | **Cluster** | **MNI coordinates** | | | ***F* values** |
| --- | --- | --- | --- | --- | --- | --- | --- |
|  |  |  | **size** | **X** | **Y** | **Z** |  |
| **sVMHC**  **dVMHC** | R/L Cerebelum_6  R/L IOG  R/L SOG  R/L IOG | /  19  18  19 | 27  34  27  10 | ±12  ±33  ±24  ±51 | -81  -84  -93  -75 | -15  -3  12  -9 | 13.6945  14.7857  12.6648  29.0878 |
| **sDC** | L MOG | 18 | 19 | -18 | -99 | 6 | 12.0143 |
|  | R MOG | 18 | 10 | 33 | -81 | 9 | 13.9386 |

Note: GRF correction, voxel level: *p* < 0.001, cluster level: *p* < 0.05

Abbreviations: BA, Brodmann’s areas; dVMHC, dynamic voxel-mirrored homotopic connectivity; IOG, inferior occipital gyrus; L, left; MNI, Montreal Neurological Institute; MOG, middle occipital gyrus; R, right; SOG, superior occipital gyrus; sVMHC, static voxel-mirrored homotopic connectivity.

**Validation analyses**

To confirm our main findings of the dVMHC alterations, two different sliding-window lengths [30 TR (60 s) and 70 TR (140 s)] were used to perform further validation analyses.

**Validation results**

Analyses using different sliding-window lengths (30 TRs and 70 TRs) supported our main findings of the dVMHC alterations (**Table S2** and **Figure S1**).

**Table S2.** Brain areas showing significant differences in dVMHC values across the three groups with different sliding-window lengths (30 TRs and 70 TRs).

| **Conditions** | **Brain region** | **BA** | **Cluster** | **MNI coordinates** | | | ***T* values** |
| --- | --- | --- | --- | --- | --- | --- | --- |
|  |  |  | **size** | **X** | **Y** | **Z** |  |
| **30 TRs** |  |  |  |  |  |  |  |
| DON vs non-DON | R/L IOG | 19 | 10 | ±51 | -72 | -9 | -7.3098 |
| **70 TRs** |  |  |  |  |  |  |  |
| DON vs non-DON | R/L IOG | 19 | 7 | ±51 | -72 | -9 | -6.5139 |

Note: GRF correction, voxel level: *p* < 0.001, cluster level: *p* < 0.05

Abbreviations: BA, Brodmann’s areas; dVMHC, dynamic voxel-mirrored homotopic connectivity; IOG, inferior occipital gyrus; L, left; MNI, Montreal Neurological Institute; R, right.

**Figure S1.** Brain areas showing significant differences in dVMHC values among DON, non-DON and HC groups with different sliding-window lengths (30 TRs and 70 TRs) (voxel level: *p* < 0.001, cluster level: *p* < 0.05, GRF corrected). In the ANCOVA results, red denotes regions that differed among the three groups. In the post-hoc pairwise comparison results, cold colors indicate decreased dVMHC values. ANCOVA, analysis of covariance; dVMHC, dynamic voxel-mirrored homotopic connectivity; DON, dysthyroid optic neuropathy; R, right; L, left.
